# Case Report: Protein-Losing Enteropathy in Association With Tuberculosis-Related Constrictive Pericarditis

**DOI:** 10.3389/fped.2022.875032

**Published:** 2022-05-30

**Authors:** Yue Xi, Zhi Chen, Kun Hao, Xiaorong Liu

**Affiliations:** ^1^Department of Nephrology, Beijing Children's Hospital, Capital Medical University, National Center for Children's Health, Beijing, China; ^2^Department of Lymphatic Surgery, Beijing Shijitan Hospital, Capital Medical University, Beijing, China

**Keywords:** protein-losing enteropathy, hypoalbuminemia, intestinal lymphangiectasia, hematuria, constrictive pericarditis

## Abstract

Protein-losing enteropathy (PLE) is a clinical disorder in which an excessive amount of serum protein is lost into the gastrointestinal tract, resulting in hypoproteinemia and malnutrition. PLE is associated with a wide range of gastrointestinal disorders and the rare complication of constrictive pericarditis. We report a case in which pericardiectomy achieved marked improvement of extremely severe hypoalbuminemia caused by PLE associated with tuberculosis-related constrictive pericarditis. The formation of diarrhea and edema was aggravated by PLE, resulting in hypoalbuminemia. Cardiac computed tomography showed a calcified pericardium. Echocardiography showed decreased cardiac function underlying PLE. Functional imaging with technetium-99m serum albumin identified the region of protein leakage as the intestine. After pericardiectomy, the diarrhea ceased completely. Serum albumin concentrations were increased (3.3–3.7 g/dL), which indicated resolution of the PLE.

## Case Report

A 14-year-old boy was admitted to our hospital because of diarrhea, hypoalbuminemia, and gross hematuria. He had been well until 5 years earlier, when he developed edema in the eyelid and lower extremities. At that time, he had a serum albumin concentration of 2.63 g/dL and 24-h urinary protein quantitation of 4,400 mg. Stool cultures were sterile, and a stool examination showed no ova or parasites. A tuberculin skin test (purified protein derivative, 5 TU) was strongly positive at the 72-h timepoint. The TSPOT.TB assay qualitative results were positive. A patchy shadow was present in a computed tomography (CT) scan of the chest. The clinical diagnosis was pulmonary tuberculosis. The patient was treated with a combination of isoniazid, rifampin, and pyrazinamide for 6 months. However, his serum albumin concentration remained at 2.5–3.6 g/dL after the therapy.

Two years before the present admission, he developed diarrhea with watery stools, but had no fever or vomiting. A physical examination showed hepatic and splenic enlargement. Laboratory tests showed that his serum albumin concentration was low at 1.75 g/dL. The qualitative results of the TSPOT.TB assay were positive. A dense consolidation was present in the right lobe with enlarged mediastinal lymph nodes, ascites, and pleural and pericardial effusions on chest CT. Cardiac CT showed a calcified cardiac base and pericardium. Echocardiography showed moderate right ventricular and auricular dilatation with pulmonary arterial hypertension. The clinical diagnoses were pulmonary tuberculosis, hypoproteinemia, and diarrhea. The patient was treated with a combination of isoniazid, rifampin, ethambutol, and pyrazinamide for 8 months. Albumin infusions were also administered. However, the albumin infusions had only short-term effects and could be used only as a bridging intervention. Serum albumin concentrations remained at 1.5–2.5 g/dL. The diarrhea was still present.

Four months before the present admission, the patient visited a local hospital because of hematuria. Persistent splenomegaly and hepatomegaly were found. The serum albumin concentration was 2.17 g/dL. Urinalysis showed a protein concentration of 220 mg/24 h and a red cell count of 8,634/μL. Cardiac CT showed a calcified pericardium. Chest CT showed right-lobe pneumonia with pleural effusion. The patient began treatment with captopril (an angiotensin-converting enzyme inhibitor) and dipyridamole. The diarrhea persisted.

**Figure 1:**
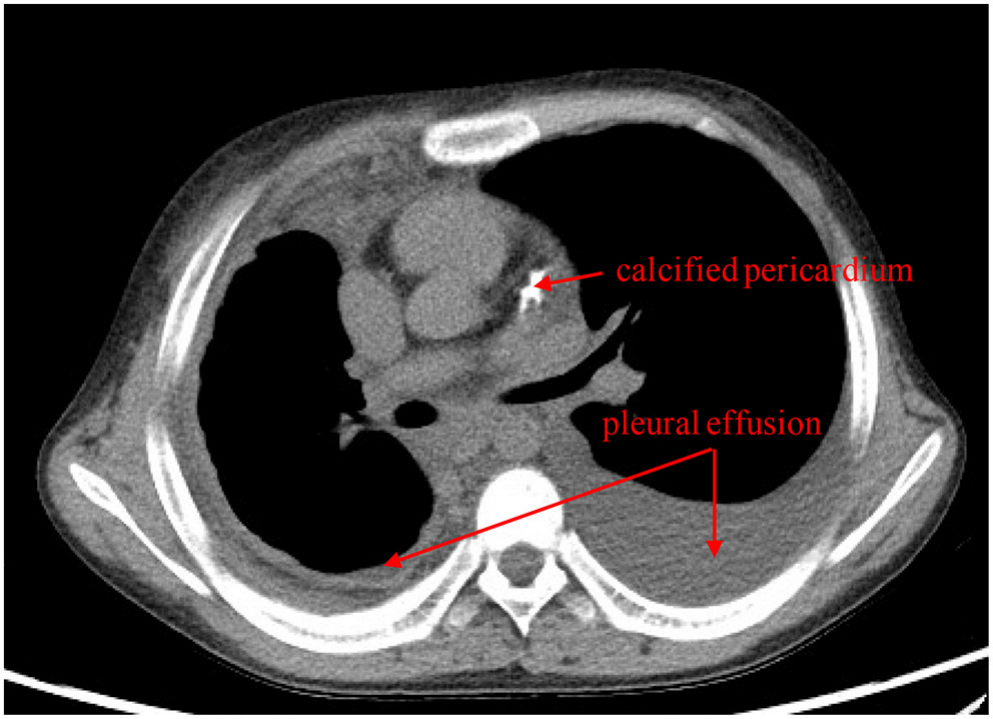
Cardiac computed tomography shows bilateral pleural effusion and a calcified pericardium (red arrows).

**Figure 2:**
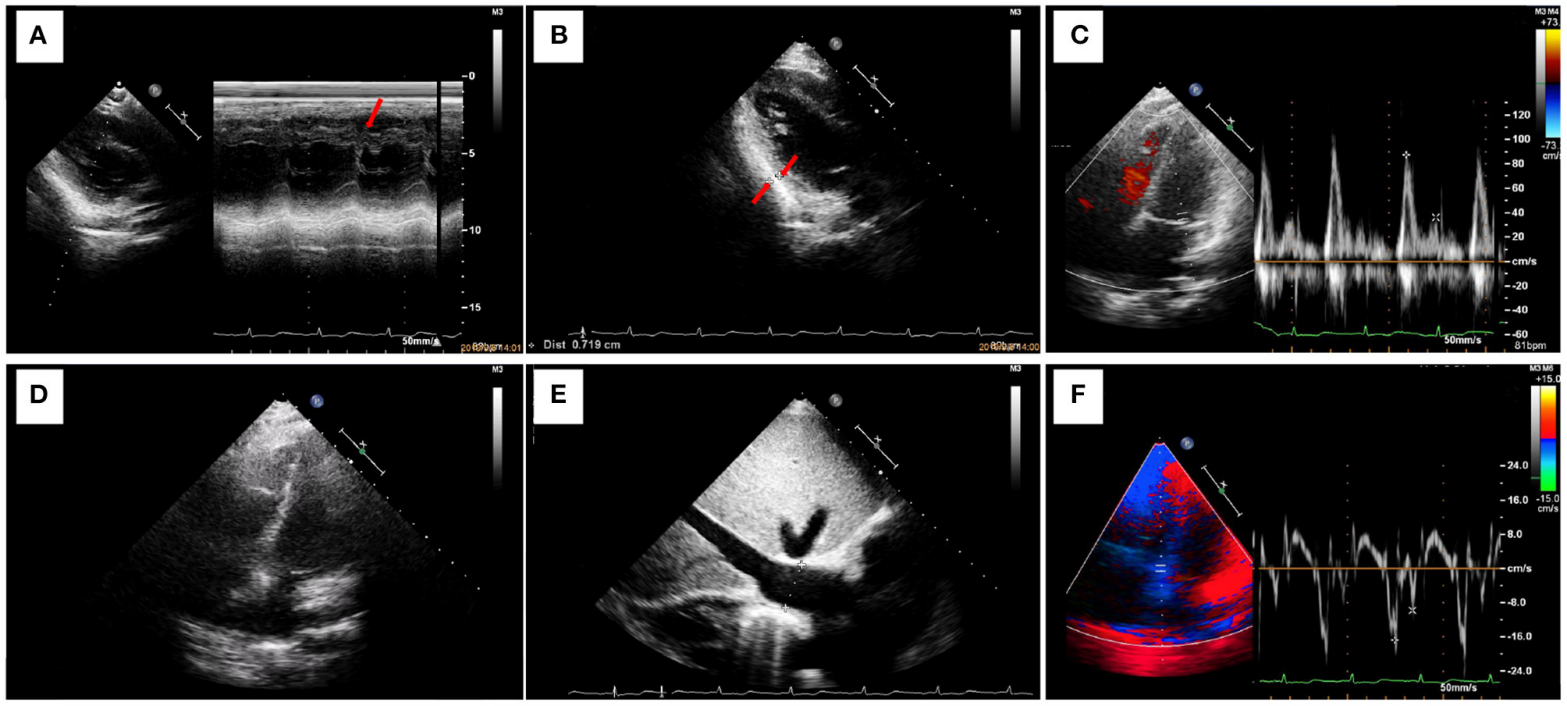
Ultrasonography shows decreased cardiac function underlying protein-losing enteropathy. **(A)** M-mode ultrasonography shows thickening of the posterior pericardium of the posterior wall of the left ventricle and signs of a septal notch. The left ventricular ejection fraction is 77%. **(B)** The posterior pericardium of the posterior wall of the left ventricle is thickened (thickness, 7 mm). **(C)** Pulse Doppler shows limited ventricular diastolic activity. The high peak E and low peak A result in an E/A of >2. **(D)** A four-chamber view of the heart shows marked thickening of the pericardium and atrial enlargement. **(E)** The inferior vena cava is greatly widened (width, 26 mm). **(F)** Myocardial relaxation is normal.

**Figure 3:**
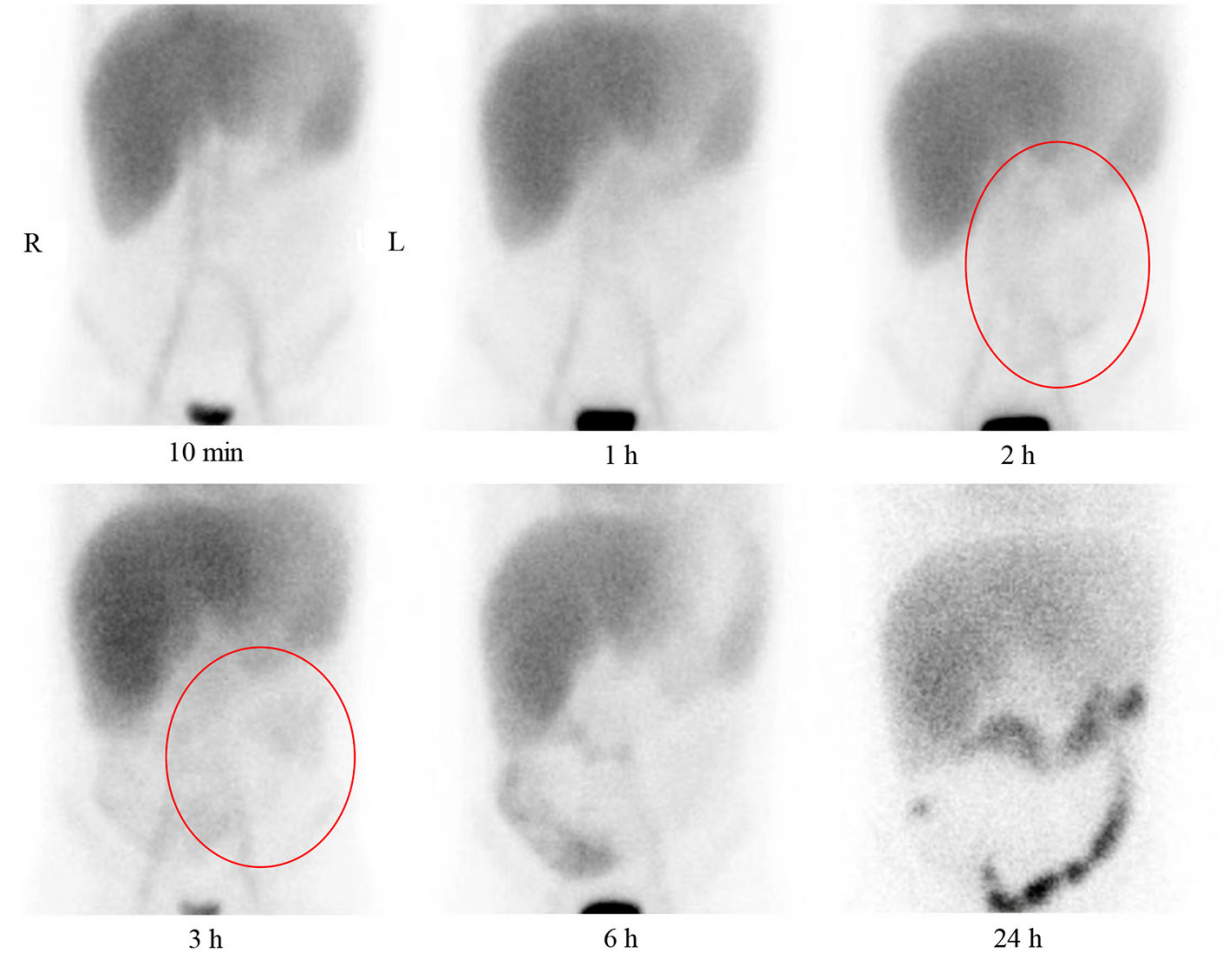
Anterior plane abdominal scintigraphy at 10 min and 1, 2, 3, 6, and 24 h after intravenous administration of technetium-99m-labeled human serum albumin. Tracer accumulation in large vessels, heart blood pool, liver, and kidney is present at 10 min and 1 h. Tracer accumulation in the abdomen is not present at 10 min and 1 h. Tracer accumulation in the lower abdomen is vaguely observed at 2 and 3 h (red circles). A large amount of tracer accumulation is present in the right lower abdomen at 6 h, and the radionuclide shows the bowel. Clear colonic tracer accumulation is present at 24 h.

**Figure 4:**
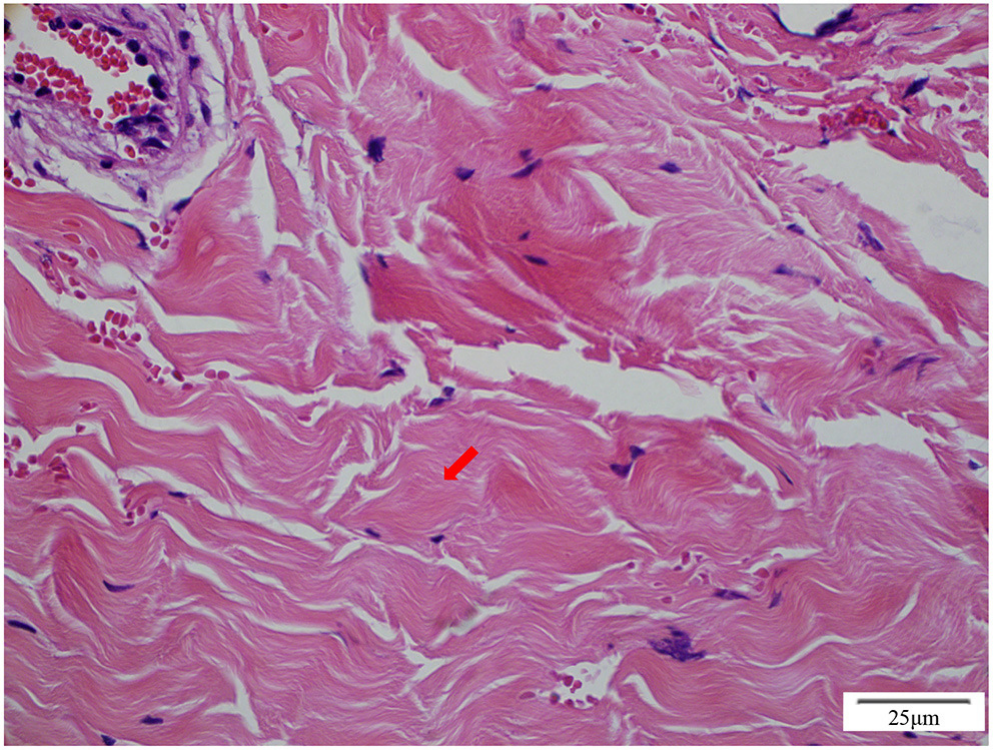
Pathological examination of the pericardium shows hyaline degeneration (red arrow).

On admission to our hospital, the patient had a normal temperature, pulse rate, respiratory rate, and blood pressure. A physical examination showed eyelid edema and jugular vein engorgement. The lungs were clear. The heart sounds were slightly weak without murmurs. Palpable splenomegaly and hepatomegaly were found. The urine was positive for protein (+), with a protein concentration of 160 mg/24 h. The sediment contained 100–200 red cells/high-powered field. The patient had pulmonary tuberculosis. Cardiac CT showed a calcified pericardium (Figure 1). Echocardiography showed decreased cardiac function underlying protein-losing enteropathy (PLE; Figure 2). The inferior vena cava was widened. Ultrasonographic examination findings of the urinary tract were normal. Functional imaging with technetium-99m serum albumin identified the region of protein leakage as the intestine (Figure 3). A biopsy of the liver showed fibrotic changes. On the basis of these findings, the patient was diagnosed with tuberculosis-related constrictive pericarditis, which was considered to have caused the PLE. Pericardiectomy was then performed. A pathological examination of the pericardium showed hyaline degeneration (Figure 4).

**Figure 5:**
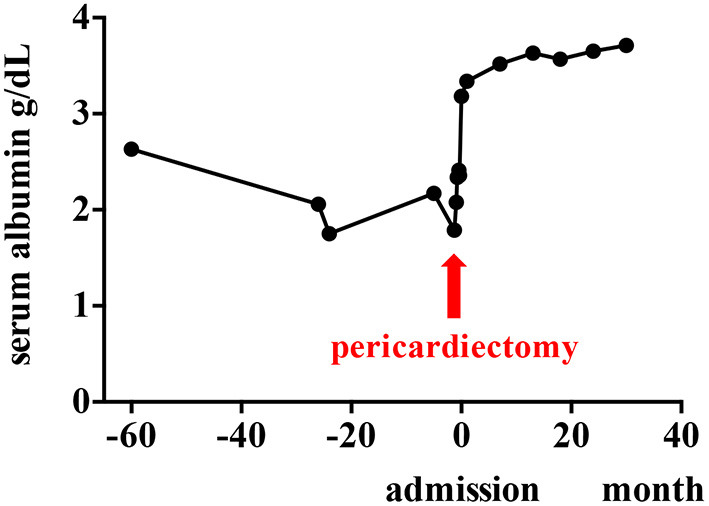
Clinical course of the patient. Serum albumin concentrations are improved after pericardiectomy.

The diarrhea ceased completely soon after the pericardiectomy. The patient's cardiac condition then improved. During a 2-year follow-up, the patient reported no symptoms, edema of the extremities, or pleural effusion. His serum albumin concentrations were increased (3.3–3.7 g/dL), which indicated resolution of the PLE (Figure 5).

## Discussion

PLE is a rare, but well-known, complication of constrictive pericarditis (1). Considerable intestinal loss of protein leads to hypoalbuminemia, which aggravates the extravascular fluid overload. The exact pathophysiological relationship between constrictive pericarditis and PLE remains obscure. In the present case, pulmonary tuberculosis led to constrictive pericarditis. A high venous pressure has been hypothesized to increase the hydrostatic pressure in the thoracic duct and presumably leads to diminished lymphatic drainage in the intestine. Notably, however, most patients with elevated venous pressure do not develop PLE (2). Most reports describe the resolution of PLE after pericardiectomy (1, 3). However, more research is required to determine the relationship between constrictive pericarditis and PLE.

Previous case reports have described postoperative improvements in the serum albumin concentration (4). However, the decision to operate requires a risk/benefit analysis regarding the expected outcome of pericardiectomy in the presence of hypoalbuminemia. This analysis is required because hypoalbuminemia has also been identified as an independent predictor of mortality after pericardiectomy (5). Although our patient had extremely severe hypoalbuminemia (1.75 g/dL) compared with previously reported cases, his serum albumin concentration increased to almost the normal range after pericardiectomy. Our results and previous data suggest that patients with constrictive pericarditis can be successfully treated with pericardiectomy, even if PLE has caused extremely severe hypoalbuminemia.

Besides the loss of serum protein into the gastrointestinal tract, other mechanisms that induce hypoalbuminemia should be considered. These mechanisms include hemodilution, liver dysfunction (liver cirrhosis), inflammation, malnutrition, and cachexia resulting from heart failure or non-heart failure-related illness. Pericardiectomy is effective in patients with constrictive pericarditis complicated by severe hypoalbuminemia because of PLE. However, the ultimate decision to operate should be made cautiously by the whole healthcare team comprising gastroenterologists, hepatologists, and cardiovascular surgeons.

## Data Availability Statement

The original contributions presented in the study are included in the article/supplementary material, further inquiries can be directed to the corresponding author.

## Author Contributions

YX and XL examined the patient, analyzed the laboratory and histopathology data, and discussed the case with the relevant consulting physicians. YX was the major contributor to drafting of the manuscript. ZC provided a supervising and editorial role. KH provided the functional imaging with technetium-99m serum albumin with its analysis. All authors read and approved the final manuscript.

## Funding

This work was supported by the Capital Health Research and Development of Special Grant (No. 2016-2-2094), the Research on the Application of Capital Clinical Characteristics Program of Beijing Municipal Science and Technology Commission (No. Z161100000516106), the Project of Beijing Science and Technology Commission (No. D181100000118006), and the Cultivation Fund of Beijing Children's Hospital, Capital Medical University (GPQN202007).

## Conflict of Interest

The authors declare that the research was conducted in the absence of any commercial or financial relationships that could be construed as a potential conflict of interest.

## Publisher's Note

All claims expressed in this article are solely those of the authors and do not necessarily represent those of their affiliated organizations, or those of the publisher, the editors and the reviewers. Any product that may be evaluated in this article, or claim that may be made by its manufacturer, is not guaranteed or endorsed by the publisher.

## References

[1] Mller C H Globits S Glogar D Klepetko W Knoflach P Constrictive Constrictive pericarditis without typical haemodynamic changes as a cause of oedema formation due to protein-losing enteropathy. Eur Heart J. (1991) 12:1140–3. 10.1093/oxfordjournals.eurheartj.a059848 1782939

[2] Güneri S Nazli C Kinay O Mermut C Hazan E. Chylous ascites due to constrictive pericarditis. Int J Cardiac Imag. (2000) 16:49. 10.1023/a:1006379625554 10832625

[3] Nikolaidis N Tziomalos K Giouleme O Gkisakis D Kokkinomagoulou A Karatzas N. Protein-losing enteropathy as the principal manifestation of constrictive pericarditis. J General Intern Med. (2005) 20:C5–7. 10.1111/j.1525-1497.2005.0202.x 16191147 PMC1490237

[4] Meijers B Schalla S Eerens F Van Suylen RJ Broers B Cheriex EM. Protein-losing enteropathy in association with constrictive pericarditis. Int J Cardiovasc Imag. (2006) 22:389–92. 10.1007/s10554-005-9067-2 16502021

[5] Moriyama H Kohno T Nishiyama T Hattori O Maekawa Y Yoshida K. Constrictive pericarditis and protein-losing enteropathy. Circul Heart Fail. (2016) 9:e003666. 10.1161/CIRCHEARTFAILURE.116.003666 27864304

